# CYP19A1 May Influence Lambing Traits in Goats by Regulating the Biological Function of Granulosa Cells

**DOI:** 10.3390/ani12151911

**Published:** 2022-07-27

**Authors:** Yan Zhang, Xiang Chen, Zhinan Zhou, Xingzhou Tian, Peifang Yang, Kaibing Fu

**Affiliations:** 1 Key Laboratory of Animal Genetics, Breeding and Reproduction in The Plateau Mountainous Region, Ministry of Education, Guizhou University, Guiyang 550025, China; yanzhanggzu@163.com (Y.Z.); serendipityznzhou@163.com (Z.Z.); tianxingzhou@yeah.net (X.T.); peifang0626@yeah.net (P.Y.); kbinfu@163.com (K.F.); 2 Key Laboratory of Animal Genetics, Breeding and Reproduction, Guiyang 550025, China; 3 College of Animal Science, Guizhou University, Guiyang 550025, China

**Keywords:** goats, follicular granulosa cells, CYP19A1, cell proliferation, lambing traits

## Abstract

**Simple Summary:**

Aromatase (*CYP19A1*), a member of the cytochrome family, is widely expressed in ovarian and granulosa cells and is primarily responsible for the conversion of androgens to estrogens. Increased expression of *CYP19A1* in follicular granulosa cells has implications for cell proliferation, steroid hormone secretion, and the expression of related functional indicator genes. We hypothesize that *CYP19A1* may indirectly influence lambing numbers in goats by regulating follicular cell growth and development, as well as ovarian ovulation.

**Abstract:**

Abnormal expression of *CYP19A1*, a gene related to steroid hormone synthesis, causes steroid hormone disruption and leads to abnormal ovulation in granulosa cells. However, the exact mechanism of *CYP19A1* regulation is unclear. In this study, we confirmed the localization of *CYP19A1* in goat ovarian tissues using immunohistochemistry. Subsequently, we investigated the effects of *CYP19A1* on granulosa cell proliferation, steroid hormone secretion, and expression of candidate genes for multiparous traits by overexpressing and silencing *CYP19A1* in goat granulosa cells (GCs). The immunohistochemistry results showed that *CYP19A1* was expressed in all types of follicular, luteal, and granulosa cells, with subcellular localization results revealing that CYP19A1 protein was mainly localized in the cytoplasm and nucleus. Overexpression of *CYP19A1* significantly increased the mRNA levels of *CYP19A1*, *FSHR*, and *INHBA*, which are candidate genes for multiple birth traits in goats. It also promoted cell proliferation, *PCNA* and *Cyclin E* mRNA levels in granulosa cells, and secretion of estrogen and progesterone. However, it inhibited the mRNA levels of *STAR*, *CYP11A1*, and *3βSHD*, which are genes related to steroid synthesis. Silencing *CYP19A1* expression significantly reduced *CYP19A1*, *FSHR*, and *INHBA* mRNA levels in granulosa cells and inhibited granulosa cell proliferation and PCNA and *Cyclin E* mRNA levels. It also reduced estrogen and progesterone secretion but enhanced the mRNA levels of *STAR*, *CYP11A1*, and *3βSHD*. *CYP19A1* potentially influenced the lambing traits in goats by affecting granulosa cell proliferation, hormone secretion, and expression of candidate genes associated with traits for multiple births.

## 1. Introduction

Ovarian follicle cells are among the fastest-growing cells. The most prominent somatic cells in follicular cells are the granulosa and luteal cells, whose ability to proliferate and differentiate is the dominant factor in follicular development and maturation [1,2]. The ovary possesses two main steroid-producing cell types: the membrane cells responsible for androgen synthesis, and the granulosa cells responsible for converting androgens into estrogens and directing progesterone synthesis [3]. Steroid hormones play an important role in many follicle cells throughout their growth phase [4]. For instance, progesterone (P4) and estradiol (E2) are involved in regulating the cyclic derivation of ovarian follicles [5] and act as antioxidants to protect follicles from oxidative stress and atresia [6,7]. Notably, the proliferation and differentiation of granulosa cells are also essential for follicular and oocyte development, ovulation, and luteinization [8]. Follicular granulosa cells are thus of great significance to the success of early gestation. Aromatase, isolated from the placenta in 1988 [9,10], belongs to the cytochrome P450 family and is responsible for the conversion of androstenedione (A2), testosterone (T), and 16a-hydroxyandrostenediol into estrone (E1), oestradiol (E2), and estriol (E3), respectively. Zhang et al. proposed that CYP19A1 is a vital enzyme in estrogen synthesis and plays an important role in establishing and maintaining pregnancy [11]. Similarly, Vega et al. found that *CYP19A1* is also involved in regulating folliculogenesis and the behavior of follicular atresia in cattle [12], and it catalyzes the conversion of androgens to estrogens [13]. In addition, the *CYP19A1* gene also plays an important role in mammalian gonadal development [14,15]. Its presence largely affects gonadal development in the testis [16] and luteal cells [17] of horses and the genitalia [18] of pigs and fetal sheep [19,20]. Luo et al. [21] found that the *CYP19A1* gene is involved in regulating follicular cell apoptosis and ovarian damage using a mice model. Notably, *CYP19A1* is also a marker gene associated with steroids and has a dynamic indicator function in response to steroid hormone secretion [22]. Wu et al. (2022) reported that abnormal expression of *CYP19A1* in sheep follicular granulosa cells may cause disturbances in ovarian steroid hormone secretion, thereby affecting normal ovulation [23]. Based on these studies, it is evident that *CYP19A1* plays an integral role in regulating an animal’s reproductive physiology. To date, only a few studies have assessed the effects of *CYP19A1* on the biological behavior and kidding traits of goat follicular granulosa cells. In conclusion, we aimed to study the effects of *CYP19A1* on steroid secretion characteristics, reproduction-related gene expression, and granulosa cell proliferation by overexpressing and interfering with the expression of the CYP19A1 gene in goat follicle granulosa cells, so as to provide reference for improving goat reproductive performance.

## 2. Materials and Methods

### 2.1. Animal Sourcing, Cell Collection, and Cell Identification

The goat GCs were collected from the healthy ovaries of six 36-month-old Qianbei horse goats (Xishui, China, China-Fuxing Animal Husbandry Co., Ltd.), which were slaughtered by Fuxing Animal Husbandry staff. Left ovary samples were preserved in phosphate-buffered saline (PBS) amended with antibodies (penicillin and streptomycin) to culture the granulosa cells. The right ovary was fixed in 4% paraformaldehyde solution and was used for immunohistochemistry and HE staining. The granulosa cells were also identified through cellular immunofluorescence for FSHR-specific expression of protein staining [24]. All animal experiments were approved by the Animal Ethics Committee of Guizhou University (Guiyang, China).

### 2.2. Plasmid Construction

Plasmids were constructed after PCR amplification by recovering the gels to obtain pure target genes and pEGFP, which were ligated using T4 DNA ligase, transferred to DH5α receptor cells, and then shaken to extract positive plasmids. The PCR system (25 μL) comprised 1 μL of DNA template, 1 μL each of upstream and downstream primer (10 pmol/µL), 12.5 μL of Green Mix, and 9.5 μL of ddH2O. Multiple pairs of CYP19A1 interfering sequences were designed following the shRNA design principles and sent to GEMA Ltd. (Shanghai, China) for synthesis. The primers (Table 1) for the corresponding assays were sent to Bioengineering Co., Ltd. (Shanghai, China) for synthesis (Login ID: NM_001285747.1). Table 2 outlines the primers for each biological cytokine function genes (all genes Tm = 60 °C).

### 2.3. Cell Culture and Transfection

Ovary tissues were collected from healthy Qianbei Ma goats, and the granulosa cells were subsequently collected from the follicles using the follicle isolation method [25]. The follicles were first punctured using a 10-gauge needle, and the follicular fluid was drained into a culture dish containing DMEM-F/12 (gibco, Beijing, China). The collected mixture was transferred to a 10 mL centrifuge tube and centrifuged at 1000 rpm for 10 min. The supernatant was discarded, the previous step repeated, and DMEM-F/12 (gibco, Beijing, China) containing 15% fetal bovine serum and 2% penicillin–streptomycin was added to the bottom of the tube using a Bachmann pipette (Wuxi, China, NEST Biotechnology Co.,Ltd.), which was blown and mixed. The cell suspension was then transferred into a 25 cm^2^ cell culture flask (Wuxi, China, NEST Biotechnology Co., Ltd.) and cultured in a 37 °C cell incubator (Thermo Fisher Scientifific, Waltham, MA, USA). The cells were then evenly plated into six empty plates (Wuxi, China, NEST Biotechnology Co.,Ltd.) upon attaining an adherence of 80–90%, followed by the transfer of the overexpression and silencing plasmids into follicular granulosa cells using a Lipofectamine 2000 kit (Thermo Fisher Scientifific, Waltham, MA, USA) according to the manufacturer’s instructions. The cells were harvested after 48 h based on the luminescence of RNA.

### 2.4. Total RNA Extraction and Reverse Transcription

Total RNA was extracted from goat granulosa cells using TRIzol reagent (Solarbio, Beijing, China). The first-strand cDNA was subsequently synthesized using a StarScript II First-strand cDNA Kit (GenStar, Beijing, China) following the manufacturer’s instructions, and the reaction products were stored at −20 °C.

### 2.5. Real-Time PCR

The effects of the CYP19A1 gene on the expression of reproduction-related candidate genes and proliferation-related indicator genes (*BMPR-IB*, *FSHR*, *INHBA*), proliferation-related genes (*Cyclin E*, *PCNA*), and steroid hormone-related genes (*STAR*, *CYP11A1* and *3βSHD*) were determined by measuring the expression levels of these genes in goat granulosa cells on a CFX 9600 real-time PCR instrument (Bio-Rad, Hercules, CA, USA). Table 2 outlines the details of the real-time PCR primers. A 10 μL real-time PCR reaction mix (containing 5 µL of 2 × Es Taq Master Mix, 3.2 µL of RNase-free ddH_2_O, 0.5 µL of cDNA, and 0.4 µL each of forward and reverse primers (10 pmol/µL)) was prepared following the kit system and subjected to a three-step PCR program (95 °C for 2 min, 95 °C for 15 s, annealing temperature (see Table 2 for details) for 30 s, 72 °C for 30 s, with the melting curve set automatically by the machine). The PCR assay was replicated thrice for each test sample. The expression data were normalized using the expression of the β-actin we used as the internal reference. The relative changes in gene expression were then calculated using the relative quantification method 2^−^^ΔΔCT^ [26].

### 2.6. Cell Proliferation Assay

The effects of overexpression and silencing of the *CYP19A1* gene on the proliferative activity of follicular granulosa cells were detected using the CCK-8 method. Plasmids in the negative control and test groups were incubated at 37 °C and 5% CO_2_ for 6, 12, 24, and 48 h after transfection, following the instructions for the CCK-8 reagent (Beyotime Biotechnology, Shanghai, China). Their absorbance (OD) at 450 nm was subsequently measured using an enzyme marker (Thermo Fisher Scientifific, Waltham, MA, USA). The expressions of *PCNA* and *Cyclin E* proliferation markers at the transcriptional level were also measured.

### 2.7. Steroid Assay

The plasmids were seeded into 96-well plates (Wuxi, China, NEST Biotechnology Co., Ltd.) in triplicate, followed by collection of 100 µL of the supernatant from each culture medium after 6, 12, 24, and 48 h. The supernatants were stored at −80 °C.

### 2.8. Immunohistochemistry

Immunohistochemistry was performed according to a previously described protocol [24]. Ovary tissue sections (5 mm) were first fixed and embedded in paraffin. The sections were then affixed onto microscope slides (Tokyo, Japan), heated at 65 °C for 2 h, de-paraffined in xylene, and rehydrated in a series of graded ethanol. The sections were then boiled in 0.1 M citrate buffer (pH = 6.0) in a microwave oven for 15 min and then cooled to 37 °C for antigen repair. The sections were subsequently washed with PBS and incubated in 3% hydrogen peroxide for 15 min to block the endogenous peroxidase activity. The sections were further incubated in blocking buffer (5% BSA in PBS) for 15 min at 37 °C to block non-specific binding. They were then incubated with rabbit anti-CYP19A1 polyclonal antibody (plus literature) in PBS (1:3000, AiFang biological, AF04449, Beijing, China) overnight at 4 °C, washed with PBS, and then incubated with biotinylated secondary antibody for 2 h at 37 °C and horseradish peroxidase (HRP)–streptavidin for 15 min before visualization using diaminobenzidine. The non-specific rabbit immunoglobulin G (IgG) was used in place of the primary antibody as the negative control. Digital images were acquired using a microscope (Tokyo, Japan).

### 2.9. Statistical Analysis

Differences between overexpression and silencing of *CYP19A1* on the proliferation and hormone data of granulosa cells were analyzed using *t*-tests. All data are presented as means ± SD of three biological replicates and three technical replicates to ensure the accuracy of the experimental data. The mRNA levels of target genes were normalized with a housekeeping gene (*β-actin*, 139 bp) and expressed as 2^−∆∆CT^ using samples on pEGFP-N3 or sh-NC as a calibrator [24]. The significance threshold was set at *p* < 0.05.

## 3. Results

### 3.1. Granulosa Cell Identification and HE Staining of Ovarian Tissues

**Figure 1:**
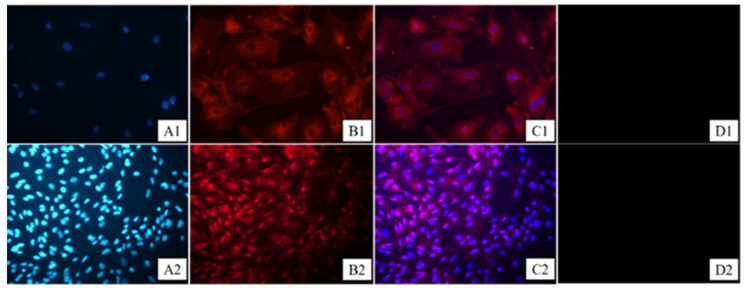
Follicular granulosa cell culture identification. (**A1**–**D1**) Immunofluorescence result of granulosa cells observed at 400×. (**A2**–**D2**) Immunofluorescence result of granulosa cells observed at 200×. (**C1**) Map combining (**A1** + **B1**), (**C2**) map combining (**A2** + **B2**), and (**D1**,**D2**) blank controls without FSHR antibody staining. (**A1**,**A2**) Granule nuclear staining, (**B1**,**B2**) cytoplasmic staining, and (**C1**,**C2**) nuclear and cytoplasmic synthesis maps.

**Figure 2:**
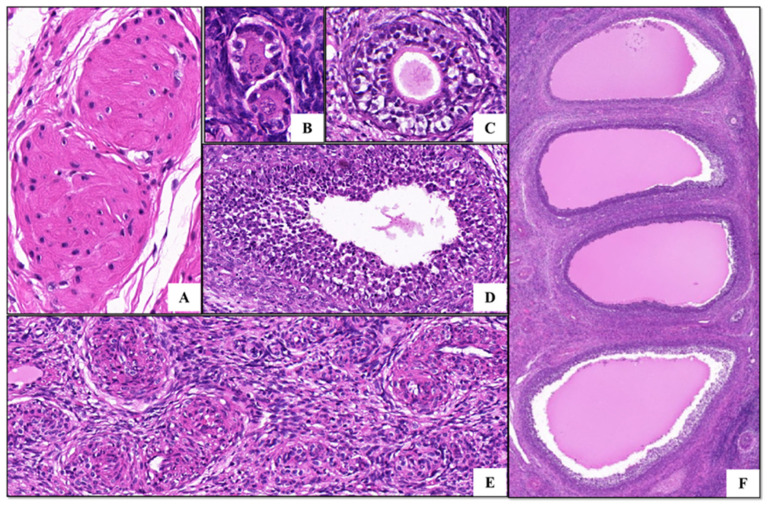
HE staining of ovarian tissue: (**A**–**D**) 400×, (**E**) 200×, (**F**) 50×. (**A**) Corpus luteum; (**B**) primordial follicle cells; (**C**) secondary follicle; (**D**) tertiary follicle; (**E**) atretic follicle; (**F**) mature large follicle group.

Follicle-stimulating hormone receptor (FSHR) is a transmembrane glycoprotein expressed in the granulosa cells of adult animals, so it can be used as a marker protein for granulosa cells. The assay uses indirect immunofluorescence to detect the purity of the FSHR protein. We found that more than 98% of the cells were stained and FSHR was located on the cell membrane, indicating that the goat primary cells were of high purity, confirming that the primary granulosa follicle cells of the Qianbei Ma goats were successfully cultured (Figure 1). The ovarian tissues of the Qianbei Ma goats were healthy and free of lesions, and had normal and clearly visible follicle morphology at all levels. Primordial, primary, and secondary follicles could be observed, allowing for the continuation of subsequent experiments (Figure 2).

### 3.2. Figures, Tables, and Schemes

**Figure 3:**
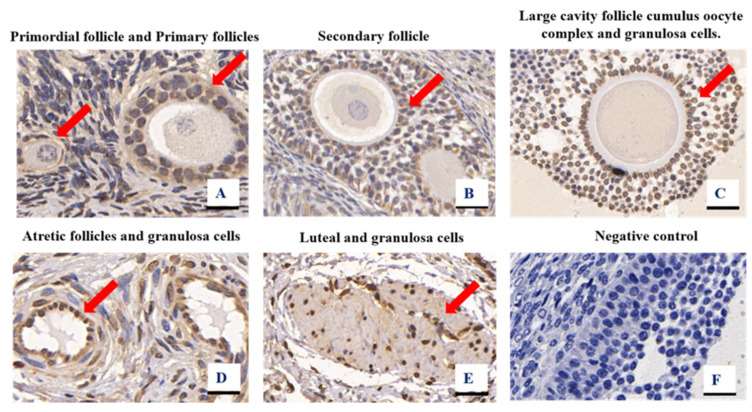
CYP19A1 protein subcellular localization results, 400×.

**Figure 4:**
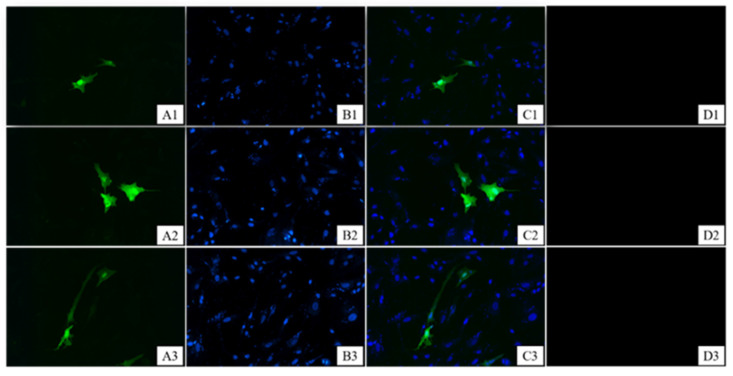
CYP19A1 protein localization, 400×. C (**C1**–**C3**) Diagram combining A (**A1**–**A3**) + B (**B1**–**B3**); (**D1**–**D3**) blank control (n = 3).

The localization of CYP19A1 expression in the ovary tissues was determined using immunohistochemistry. The CYP19A1 protein was present in the primordial follicles, secondary follicles, oocyte complexes, luteal cells, and surrounding granulosa cells of developing goat follicles (Figure 3). The control reactions of the CYP19A1 antibodies tested confirmed the absence of non-specific staining (Figure 3F), suggesting that CYP19A1 is widely distributed in all parts of the ovary. The results of subcellular localization assays further revealed that the CYP19A1 protein was localized in the cell membrane and cytoplasm of granulosa cells (Figure 4).

### 3.3. Effects of Overexpression and Silencing of CYP19A1 on the mRNA Expressions of BMPR-IB, FSHR, and INHBA in Granulosa Cells

**Figure 5:**
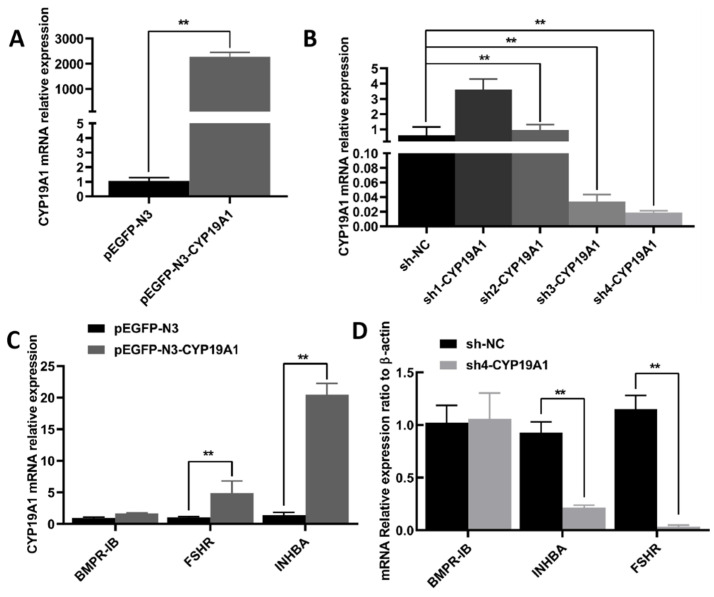
Validation of *CYP19A1* overexpression and silencing expression vector efficiency and results of genetic assays for reproduction-related indicators. (**A**) pEGFP-N3-CYP19A1 efficiency validation; (**B**) sh1-4-CYP19A1 efficiency validation; (**C**) results of overexpression of *CYP19A1*; (**D**) results of silencing *CYP19A1*. ** indicates highly significant difference (*p* < 0.01). The mRNA levels of target genes were normalized with housekeeping gene (β-actin, 139 bp) and expressed as 2^−∆∆CT^ using samples on pEGFP-N3 or sh-NC as calibrator. Results are indicated by means ± SD (n = 3).

We investigated the effects of overexpression and silencing of the *CYP19A1* gene on the mRNA levels of *BMPR-IB*, *FSHR*, and *INHBA*, genes related to multiple birth traits, in granulosa cells. We first verified the expression efficiencies of *CYP19A1* overexpression and silencing vector in granulosa cells. Overexpression of *CYP19A1* significantly increased the mRNA level of pEGFP-N3-CYP19A1 (*p* < 0.01) (Figure 5A). Among the four sequences that silenced *CYP19A1*, sh4-CYP19A1 had the best silencing efficiency (Figure 5B). Notably, overexpression of *CYP19A1* significantly increased the mRNA levels of *FSHR* and *INHBA* in granulosa cells (*p* < 0.01) (Figure 5C) and vice versa (*p* < 0.01) (Figure 5D).

### 3.4. Effects of CYP19A1 on the Proliferation of Goat Granulosa Cells

**Figure 6:**
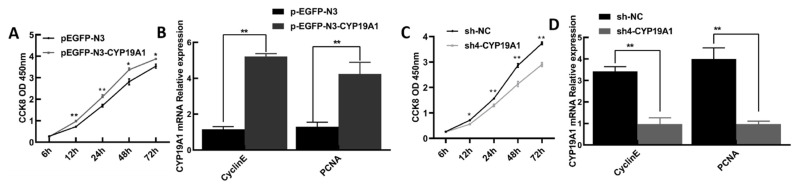
Effects of altering *CYP19A1* on proliferation of granulosa cells. (**A**) Overexpression of *CYP19A1* promotes *PCNA* and *Cyclin E* mRNA expression levels; (**B**) overexpression of *CYP19A1* promotes granulosa cell proliferation; (**C**) silencing of *CYP19A1* inhibits *PCNA* and *Cyclin E* mRNA expression levels; (**D**) silencing of *CYP19A1* inhibits granulosa cell proliferation. The mRNA levels of target genes were normalized with housekeeping gene (β-actin, 139 bp) and expressed as 2^−∆∆CT^ using samples on pEGFP-N3 or sh-NC as a calibrator. Results are indicated by means ± SD (n = 3). ** indicates highly significant difference (*p* < 0.01), * indicates significant difference (*p* < 0.05).

The CCK8 assay and detection of proliferation-related genes, *PCNA* and *Cyclin E*, were used to study the effects of *CYP19A1* on the biological behavior of granulosa cells. Overexpression of *CYP19A1* gene in granulosa cells significantly increased cell proliferation (Figure 6A) and promoted the expressions of *PCNA* and *Cyclin E* genes (*p* < 0.01) (Figure 6B). In contrast, silencing of the *CYP19A1* gene in granulosa cells significantly inhibited the proliferation of granulosa cells (*p* < 0.05) (Figure 6C) and suppressed the expressions of *PCNA* and *Cyclin E* genes (*p* < 0.01) (Figure 6D).

### 3.5. Effects of CYP19A1 on the Secretion of Steroid Hormones from Goat Granulosa Cells

**Figure 7:**
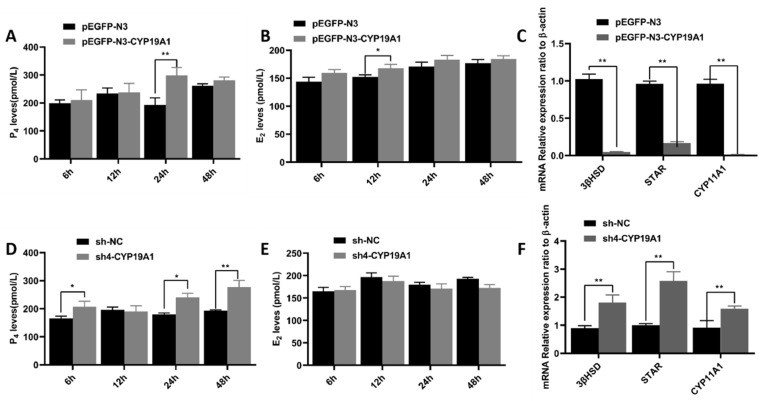
Effects of altering CYP19A1 gene on granulosa cell proliferation. (**A**) Overexpression of CYP19A1 promotes progesterone secretion in granulosa cells; (**B**) silencing CYP19A1 inhibits progesterone secretion in granulosa cells; (**C**) overexpression of CYP19A1 promotes mRNA expression levels of steroid-related genes; (**D**) overexpression of CYP19A1 promotes estrogen secretion in granulosa cells; (**E**) silencing of CYP19A1 inhibits estrogen secretion in granulosa cells; (**F**) silencing of CYP19A1 suppression inhibits the mRNA expression level of steroid-related genes. ** indicates highly significant difference (*p* < 0.01), * indicates significant difference (*p* < 0.05).

We examined the intracellular estradiol (E_2_) and progesterone (P_4_) contents 6, 12, 24, and 48 h after the introduction of *CYP19A1* into the cells to investigate the effects of altering the *CYP19A1* content on steroid hormone secretion. Overexpression of *CYP19A1* in granulosa cells increased progesterone and estrogen secretion at 6, 12, 24, and 48 h (Figure 7A,B). Similarly, the secretion of progesterone and estrogen in sh-NC was higher than that in the sh4-CYP19A1-treated group (Figure 7D,E), especially at 12, 24, and 48 h, but did not reach significant levels (Figure 7E). Similarly, overexpression of *CYP19A1* significantly down-regulated the expressions of *STAR*, *CYP11A1*, and *3βSHD* (*p* < 0.01) (Figure 7C), while silencing of *CYP19A1* significantly up-regulated the expressions of *STAR*, *CYP11A1*, and *3βSHD* (*p* < 0.01) (Figure 7F).

## 4. Discussion

Female fertility in mammals is co-determined by the number and quality of mature follicles [27]. Previously, researchers have postulated that the cytochrome P450 family is mainly expressed in mammalian theca cells, mouse granulose cells, ovarian and placental tissues, and other tissues [11,28,29]. These findings are consistent with our immunohistochemistry test results, suggesting that *CYP19A1* is located in goat follicular granulosa cells. Interestingly, *CYP19A1* is expressed in the pre-granulosa cells of female chicken embryonic gonads, unlike in mammals, where it is expressed in ovarian granulosa cells [30], a difference attributed to species differences between birds and mammals. In this study, *CYP19A1* was present in theca, luteal granulosa, and cumulus complex cells of follicles at the different stages of follicular development. The subcellular localization further revealed that *CYP19A1* might play a vital role in the nucleus and cytoplasm of granulosa cells. These findings collectively suggest that *CYP19A1* is involved in the whole process of goat follicular development, including follicular maturation, normal ovulation, and periodic follicular derivation.

It is important to understand the molecular basis of follicular development and maturation because of its significant impact on the reproductive ability of goats [3]. The medium and high single expressions of the FSHR protein receptor in granulosa cells were identified, and follicular granulosa cells were successfully cultured to analyze the effects of changes in the *CYP19A1* gene on the biological functions of granulosa cells. The growth and development of follicles in the ovary re dependent on the proliferation of granulosa cells [30]. Proliferating cell nuclear antigen (*PCNA*) is a proliferation marker widely distributed in various cells. The content of *PCNA* in cells generally reflects the proliferative rate of cells [31]. *Cyclin E* can also reflect the cell proliferative rate because it regulates the transition from the G0/G1 phase to the S phase in cells [32]. *CYP19A1* was upregulated and silenced to investigate whether the changed expression of *CYP19A1* would affect the proliferation of granulosa cells. Up-regulation of CYP19A1 improved the proliferation of granulosa cells and increased *PCNA* and *Cyclin E* expressions (*p* < 0.01). However, silencing of *CYP19A1* decreased the proliferative rate of granulosa cells and the mRNA levels of *PCNA* and *Cyclin E* genes (*p* < 0.01). These findings indicated that the alteration in the *CYP19A1* content in granulosa cells significantly affected the proliferative rate of granulosa cells and the mRNA expressions of *INHBA* and *FSHR*.

*FSHR* is a member of the transforming growth factor-β (TGF-β) family and regulates biological behaviors together with multiple cytokines, including regulating cell proliferation, apoptosis, and differentiation. It is mainly expressed in granulosa cells [33]. Relevant *FSHR* studies have indicated that mutation of the 5′UTR region in this gene can improve the litter size of both small-tailed Han sheep and Hu sheep [34,35]. The *FSHR* gene is a member of the G protein-coupled receptor family and is only expressed in mammalian follicular granulosa cells [36]. Its expression is increased with the growth of healthy follicles [37], playing a core role in promoting follicular maturation and increasing ovulation. The *INHBA* gene (Inhibin (INH) βA subunit) is a marker gene of granulosa cell development. It is expressed in oocytes of all follicle types, granulosa cells after the primary follicular phase, theca cells of sinus follicles, and surface epithelial cells of corpus luteum and ovaries. The expression of *INHBA* is also detected in the cumulus and mural granulosa cells in sinus follicles [38]. Li et al. postulated that *INHBA* is expressed at different follicular stages and has extremely high expression in the GCs growth period in multiparous-lamb goat population [39]. Notably, mutations in the *INHBA* gene have a significant effect on the litter size of sheep [34], thus further verifying that it can affect the lambing performance of goats by regulating the development of follicles.

Androstenedione and androgen can be transformed into estradiol and estrone with the action of aromatase in granulosa cells. Small amounts of the secreted estrogen act on ovary development, and the balance is released into the blood circulation and enters the target tissues, including the uterus, breast, and kidneys [40]. The destruction of the aromatase gene *CYP19A1* in mice results in estrogen deficiency, leading to the normal development of ovarian follicles [21]. CYP19A1 is the key signal enzyme for estradiol synthesis [41]. The content of *CYP19A1* has thus been determined in many studies to reflect the expression levels of steroid hormones. The higher the *CYP19A1* content, the higher the hormone secretion. Padmanabhan et al. postulated that a decrease in *CYP19A1* expression in the follicular granulosa cells of adult female sheep might destroy the balance between androgen and estrogen in follicles [22]. This imbalance potentially causes long-lasting follicles, which seriously endanger the periodic derivation of follicles and normal ovulation, causing low fecundity. The cholesterol side-chain lyase, which is encoded by *CYP11A1*, can catalyze the synthesis of pregnenolone, the common precursor of all steroid hormones, which plays a key role in the initial and rate-limiting steps of transformation [42]. The steroidogenic acute regulatory protein (STAR), located in the mitochondrial membrane, regulates the speed of cholesterol transport from the outer mitochondrial membrane to the inner membrane. *CYP11A1* then converts the cholesterol on the inner membrane into pregnenolone, which is the substrate for progesterone synthesis [43]. Steroidogenic acute regulatory protein (STAR) and 3-β-hydroxysteroid dehydrogenase (3βHSD) are jointly involved in the development of oocytes and significantly affect oocyte quality and pregnancy results [44,45,46]. Notably, the secretion of P_4_ and E_2_ can be improved or inhibited to a certain extent by up-regulating the *CYP19A1* gene in granulosa cells. In this study, up-regulation of *CYP19A1* in granulosa cells increased the secretion of estrogen and progesterone but down-regulated the mRNA level of enzymes related to steroid hormone synthesis, including STAR, CYP11A1, and 3βSHD (*p* < 0.01). STAR, CYP11A1, and 3βSHD are the precursors for the synthesis of testosterone and progesterone in the steroid hormone synthesis pathway, and CYP19A1 is the key enzyme for the downstream hormone synthesis [47]. *CYP19A1* inhibits the expression of *STAR*, *CYP11A1*, and *3βSHD* after excessive secretion of estrogen and progesterone in the body through negative feedback regulation, thus stabilizing hormone synthesis in the body. Similarly, the mRNA levels of the *STAR*, *CYP11A1*, and *3βSHD* genes are enhanced through negative feedback regulation (*p* < 0.01) when there is insufficient secretion of estrogen and progesterone in the body, further stabilizing hormone secretion. The findings reported herein will help us to understand the effects of the important pathway of *CYP19A1* on hormone synthesis in granulosa cells.

## 5. Conclusions

Overexpression of *CYP19A1* significantly increased the mRNA levels of *CYP19A1*, *FSHR*, and *INHBA*, which are candidate genes for multiple birth traits in goats. We found that overexpression of *CYP19A1* in granulosa cells can promote the proliferation of granulosa cells; promote the expression of *INHBA* and *FSHR* genes in granulosa cells; promote the secretion of steroid hormones (E2 and P4), inhibit the expression of the key steroid synthesis genes *STAR*, *CYP11A1*, and *3βSHD*; and can promote the expression of *INHBA* and *FSHR* in granulosa cells. Interfering with the expression of *CYP19A1* can have the opposite result of overexpression. Therefore, we speculate that the *CYP19A1* gene can regulate granulosa cell proliferation, possibly through negative feedback regulation of steroid hormone synthesis, and indirectly regulate goat follicle development and ovulation by promoting the expression of *INHBA* and *FSHR*.

## Figures and Tables

**Table 1:** CYP19A1 shRNA and CYP19A1 pEGFP primer information.

Gene Name	Primer Sequences (5′-3′)
sh1-CYP19A1	F: CACCGCGGTCACCAACATAATCAGCTTCAAGAGAGCTGATTATGTTGGTGACCGCTTTTTTG R: GATCCAAAAAAGCGGTCACCAACATAATCAGCTCTCTTGAAGCTGATTATGTTGGTGACCGC
sh2-CYP19A1	F: CACCGACCAGAATATAGGTTTCAATTTCAAGAGAATTGAAACCTATATTCTGGTCTTTTTTG R: GATCCAAAAAAGACCAGAATATAGGTTTCAATTCTCTTGAAATTGAAACCTATATTCTGGTC
sh3-CYP19A1	F: CACCGAGGCAATGATGAGGGAAATCTTCAAGAGAGATTTCCCTCATCATTGCCTCTTTTTTG R: GATCCAAAAAAGAGGCAATGATGAGGGAAATCTCTCTTGAAGATTTCCCTCATCATTGCCTC
sh4-CYP19A	F: CACCGCTGTGCAGAAAGTATGAAAATTCAAGAGATTTTCATACTTTCTGCACAGCTTTTTTG R: GATCCAAAAAAGCTGTGCAGAAAGTATGAAAATCTCTTGAATTTTCATACTTTCTGCACAGC
sh-NC	F: CACCGTTCTCCGAACGTGTCACGTTTCAAGAGAACGTGACACGTTCGGAGAATTTTTTG R: GATCCAAAAAATTCTCCGAACGTGTCACGTTCTCTTGAAACGTGACACGTTCGGAGAAC
pEGFP-N3-CYP19A1	(BglII) F: CGTCAGATCCGCTAGCGCTACCGGACTC**AGATCT**ATGCTTTTGGAAGTGCTGAAC (SacIIR): CACCATGGTGGCGATGGATCCCGGGC**CCGCGG**CGCACTCGAGGCACTTGTCTGAATTTCTC

The pEGFPpEGFP-N3-CYP19A1 vector sequence is shown in red for the homology arm, bold black for the digestion site, and black for the target fragment.

**Table 2:** Related gene real-time fluorescent primer information.

Gene Name	Primer Sequences (5′-3′)	Login ID	Fragment Size (bp)
*BMPR-1B*	F: GCTCTTGGTCCTCATCATTTTATTC R: ATGTAAGTTTCGTCCTGTTCTAACC	NC_022298.2	143
*FSHR*	F: TGTTATGTCCCTCCTTGTGCTC R: CGCTTGGCTATCTTGGTGTCA	NM_001285644.1	122
*PCNA*	F: GTAGCCGTGTCATTGCGACTCC R: GCTCTGTAGGTTCACGCCACTTG	XM_005688167.3	145
*INHBA*	F: AAGGTGGTGGATGCTCGAAA R: GTCTCCTGACACTGCTCACA	NM_001285581.1	125
*CYP19A1*	F: CCCAAGGCATTACAATGT R: TAAGGGTTTCCTCTCCAC	NM_001314145.1	169
*STAR*	F: GGTCCCCGAGACTTTGTGAG R: AATCCACTTGGGTCTGCGAG	XM_013975437.2	262
*3βHSD*	F: AGACCAGAAGTTCGGGAGGAA R: TCTCCCTGTAGGAGTTGGGC	NM_001285716.1	292
*CYP11A1*	F: CTCCAGAGGCAATAAAGAA R: TCAAAGGCAAAGTGAAACA	NM_001287574.1	145
*Cyclin E*	F: GATGTCGGCTGCTTAGAAT R: CACCACTGATACCCTGAAAC	XM_018062248.1	104
*β*-actin	F: AGATGTGGATCAGCAAGCAG R: CCAATCTCATCTCGTTTTCTG	NM_001297986.1	139

## Data Availability

The data presented in this study are available on request from the corresponding author.
